# Two effective cases of additional pedal artery angioplasty for severe lower limb ischemia following acute thrombotic artery occlusion with hypercoagulable state diseases

**DOI:** 10.1186/s42155-020-00166-7

**Published:** 2020-09-28

**Authors:** Keisuke Shoji, Kan Zen, Kenji Yanishi, Noriyuki Wakana, Naohiko Nakanishi, Takeshi Nakamura, Satoaki Matoba

**Affiliations:** Department of Cardiovascular Medicine, Kyoto Prefectural University of Medicine, 465 Kajii-cho Kawaramachi-Hirokoji, Kamigyo-ku, Kyoto, 602-8566 Japan

**Keywords:** Pedal artery angioplasty, Thrombotic artery occlusion, Hypercoagulable state disease, Critical limb ischemia, Acute limb ischemia

## Abstract

**Background:**

Acute limb ischemia (ALI) and critical limb ischemia (CLI) following ALI are life-threatening diseases. The rare potential causes of ALI include hypercoagulable state diseases, such as antiphospholipid syndrome (APS) and essential thrombocythemia (ET). Hypercoagulability often make revascularization for arterial occlusion, especially associated with infrapopliteal lesions, difficult. This is because the vessels have poor run-off, and elevated peripheral vascular resistance associated with microcirculation failure, due to a high thrombus burden. There is no established treatment for this issue.

**Case presentation:**

A 45 years-old and a 56 years-old male suffered from thrombotic arterial occlusion as a first manifestation of APS and ET, respectively. Combination therapy with aggressive anti-thrombotic therapy and revascularization, such as endovascular therapy and surgical thrombectomy based on the angiosome concept, was performed. However, the high thrombus burden caused a poor pedal outflow, and significant limb ischemia remained. Additional pedal artery angioplasty was performed to improve residual limb ischemia in each case and provided sufficient blood flow to the foot.

**Conclusion:**

The pedal artery angioplasty for thrombotic pedal artery occlusion cases, associated with hypercoagulable state diseases, seems to be a treatment option for relieving residual limb ischemia.

## Introduction

Acute limb ischemia (ALI) and critical limb ischemia (CLI) following ALI are life and limb-threatening diseases that lead to rest-pain, ischaemic ulcers, and gangrene due to impaired perfusion in the lower limbs. A rare potential cause of ALI includes thrombotic artery occlusion associated with hypercoagulable state diseases, such as antiphospholipid syndrome (APS) and essential thrombocythemia (ET) (Suzuki et al. 2016; Moulinet et al. 2016; Chong et al. 2016; Morata Barrado et al. 2009). Successful revascularisation, including thrombectomy, bypass surgery, endovascular treatment, or hybrid treatment (surgical revascularization and endovascular treatment) for thrombotic artery occlusion in hypercoagulable state patients, has been reported (Moulinet et al. 2016; Chong et al. 2016; Morata Barrado et al. 2009). However, revascularization in infrapopliteal lesions, including below-the-ankle (BTA) lesions, may be difficult because of poor run-off vessels, and elevated peripheral vascular resistance associated with microcirculation failure, due to a high thrombus burden. Herein, we describe two cases in which additional pedal artery angioplasty was effective in thrombotic artery occlusion associated with hypercoagulable state diseases.

## Case report

### Case 1

**Fig. 1:**
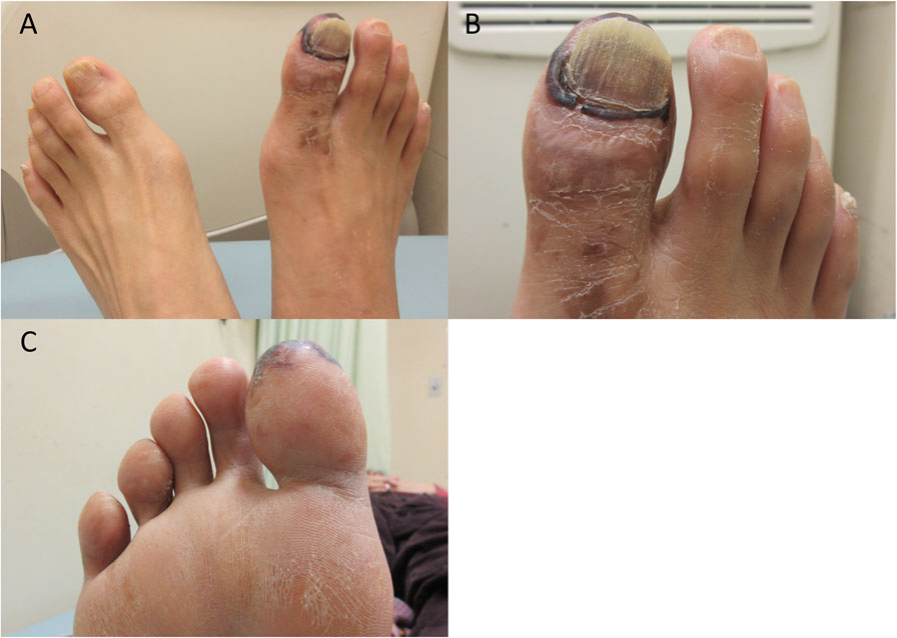
Findings of the lower limb in the case 1. **a-c** His right foot was poorly colored, and the first toe showed highly cyanosis

**Fig. 2:**
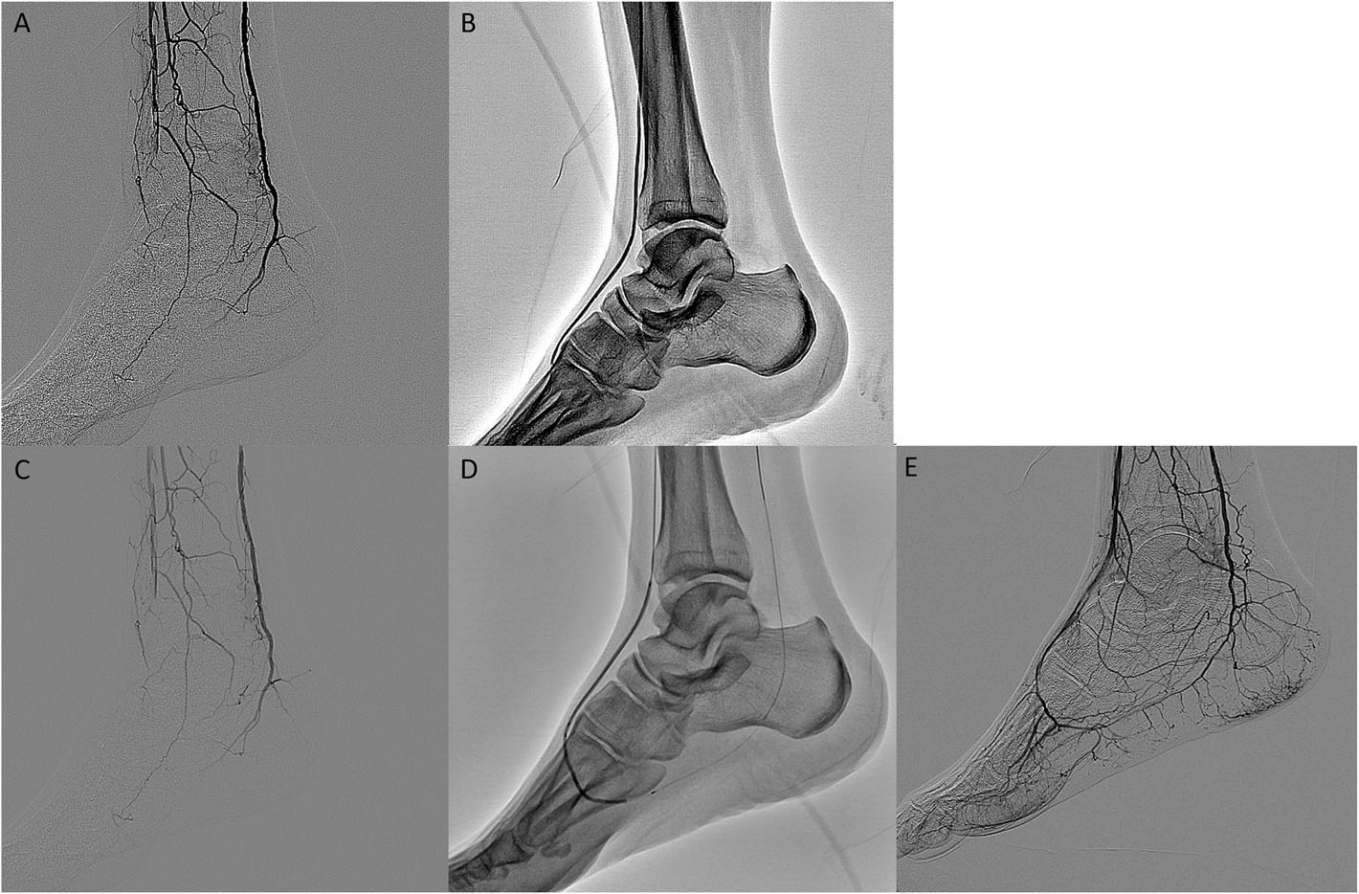
Additional pedal artery angioplasty in case 1. **a** Baseline angiography showing total occlusion from the anterior tibial artery (ATA) to the dorsalis pedis artery (DPA) in our hospital. **b** The balloon was dilated from the DPA to the ATA. **c** Angiography showed insufficient blood flow to the forefoot after ballooning on the DPA and ATA. **d** Additional balloon angioplasty was performed in the occluded pedal artery. **e** Sufficient blood flow was obtained after additional pedal artery angioplasty

A 45-year-old male who had no medical history suffered from sudden-onset rest-pain on his right limb with coldness and cyanosis 1 month ago, and his symptoms kept worsening. Computed tomography angiography revealed total occlusion in his right popliteal artery. Aggressive antithrombotic therapy (cilostazol 200 mg, warfarin 3.5 mg, prasugrel 3.75 mg, systemic urokinase infusion) and two revascularization procedures, with surgical thrombectomy and balloon angioplasty for infrapopliteal lesion via right common femoral artery, were performed. However, the improvement of peripheral perfusion below the knee was insufficient. He was diagnosed with APS from blood examination results (anti-cardiolipin β2-glycoprotein I complex antibody positive). He was referred to our hospital because of severe rest-pain and cyanosis persisting despite aggressive anti-thrombotic therapy and analgesic (acetaminophen 1950 mg, and tramadol 225 mg) (Fig. 1a-c). Therefore, we attempted to perform endovascular revascularization again. A 4.5-Fr guiding catheter (Parent Plus, Medikit, Tokyo, Japan) was inserted via left femoral artery and advanced to right distal superficial femoral artery. A 0.014-in. guidewire (Cruise, Asahi Intecc, Aichi, Japan) was advanced into the dorsalis pedis artery (DPA) with microcatheter support (Prominent BTA, Tokai Medical Products, Aichi, Japan) via a contralateral approach and a 2 mm balloon catheter was dilated from the anterior tibial artery (ATA) to the DPA, but direct flow to the forefoot could not be obtained (Fig. 2a-c). Therefore, we performed additional pedal artery angioplasty with a 2 mm balloon and could obtain direct flow to the forefoot (Fig. 2d, e). After the procedure, he was completely released from severe rest-pain and discharged from our hospital without any complications. His ischemic rest pain has not recurred since then.

### Case 2

**Fig. 3:**
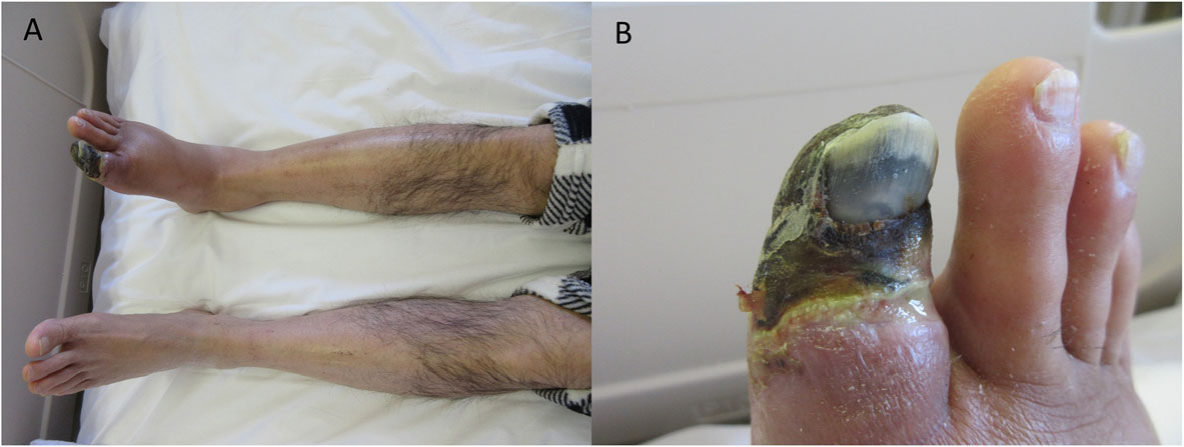
Findings of the lower limb in the case 2. **a, b** His right foot had gangrene on his first toe and was accompanied by edema and poor color

**Fig. 4:**
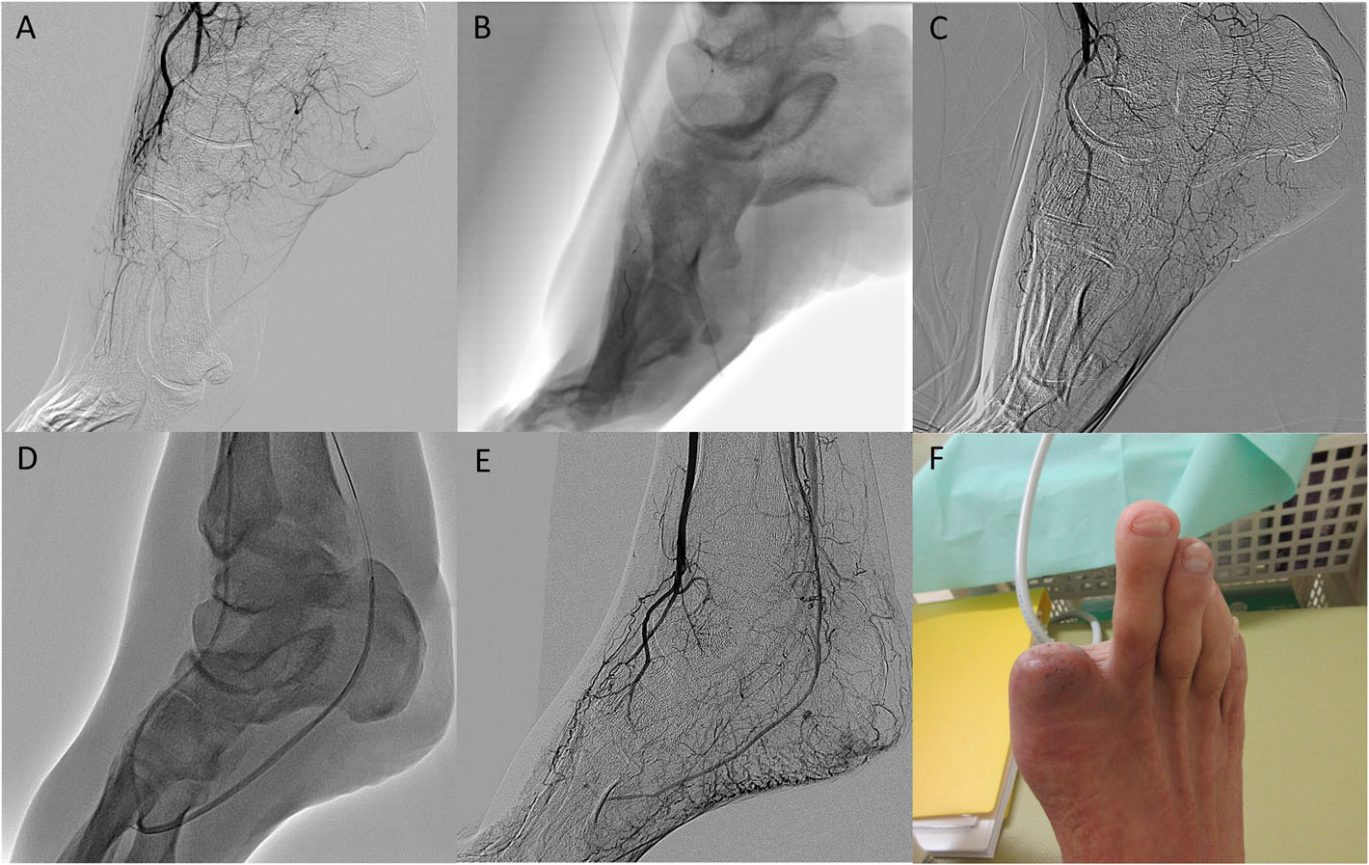
Additional pedal artery angioplasty in case 2. **a** Baseline angiography showing total occlusion in the pedal artery lesion in another hospital. **b** Balloon dilated in the dorsalis pedis artery (DPA) and digital artery forward first toe. **c** Final angiography showed insufficient revascularization in another hospital. **d** Pedal artery angioplasty was performed in our hospital. **e** Antegrade blood flow from the bilateral tibial arteries forward his foot was obtained. **f** His wound completely healed

A 56-year-old male with no medical history suffered from sudden-onset rest-pain about 3 months ago. Peripheral vasodilators agents did not improve his symptoms, and the gangrene in his first toe had been progressing (Fig. 3a, b). Angiography showed total occlusion in the right DPA and posterior tibial artery distal part, but balloon angioplasty for the DPA failed technically in another hospital (Fig. 4a-c). He was referred to our hospital for symptom-improvement and avoiding major amputation. He was diagnosed with ET with Janus kinase 2 (JAK2) V617 mutation from abnormal platelet count elevation (113.8 × 10^4^ / μL) and bone marrow biopsy findings. Combination medical therapy with cytoreduction therapy and anti-thrombotic therapy (aspirin 100 mg and rivaroxaban 15 mg) was started and pain management was enhanced (pregabalin 150 mg, acetaminophen 1600 mg, and tramadol 150 mg), but severe ischemic rest-pain persisted. Moreover, the skin perfusion pressure (SPP) around the wound could not be measured due to pain, and the SPP value near the ankle was 48 mmHg, which was insufficient for wound healing. Therefore, we attempted to perform additional pedal artery angioplasty. A 4.5-Fr guiding catheter (Parent Plus, Medikit, Tokyo, Japan) was inserted via right femoral artery and advanced to popliteal artery. A 0.014-in. guidewire was advanced into the occluded pedal artery under IVUS guidance with a bidirectional approach from both tibial arteries. First, a Jupiter FC guidewire (Boston Scientific, Tokyo, Japan) was advanced from the posterior tibial artery (PTA) to the pedal artery using a microcatheter (Prominent BTA, Tokai Medical Products, Aichi, Japan), but it could not pass into the DPA. Finally, the Jupiter FC guidewire was successfully passed from the ATA to the PTA through the pedal artery. A 2 mm balloon was dilated in the pedal artery (Fig. 4d). Successful pedal artery angioplasty improved blood flow forward to his forefoot via the bilateral tibial arteries (Fig. 4e). His rest-pain completely disappeared and the dorsal SPP value was elevated (78 mmHg). He underwent minor amputation of the necrotic part of his right first toe, and his wound was completely healed without delay (Fig. 4f).

## Discussion

We encountered two severe limb ischemia cases following acute thrombotic pedal artery occlusion, associated with hypercoagulable state diseases. The additional pedal artery angioplasty was remarkably effective in improving ischemic symptoms in these two cases.

APS is a systemic autoimmune disease characterised by recurrent venous or arterial thrombosis and recurrent foetal loss alongside the presence of antiphospholipid antibodies (Asherson et al. 1996). Moreover, ET is a chronic myeloproliferative neoplasm of unknown cause characterised by an increased number of platelets. ET provokes thrombosis and haemorrhaging as typical vascular complications (Schafer 2006). ALI in thrombotic conditions such as APS and ET is rare, but often threatens limb viability (Hinojosa et al. 2017; Ali et al. 2009). Sub-acute or chronic limb ischaemia following sudden thrombotic artery occlusion can also be a serious state, and ischemic symptoms gradually worsen (Patel et al. 2003). Therefore, revascularization should be performed with surgical and/or endovascular treatment to prevent limb amputation in ALI and CLI following ALI.

Surgical thrombectomy using a Fogarty catheter is a feasible treatment for ALI (Fogarty 2009). Moreover, hybrid treatment combined with surgical thrombectomy and additional aspiration, balloon dilation, and/or stenting under fluoroscopic guidance was recommended (de Donato et al. 2014). Catheter-directed thrombolysis, which is directly delivered within the thrombus in femoropopliteal lesions, is also a feasible treatment for ALI patients (Byrne et al. 2014). These treatment strategies had been adapted in ALI patients associated with hypercoagulable state diseases in previous reports (Moulinet et al. 2016; Chong et al. 2016; Morata Barrado et al. 2009). However, high thrombus burden associated with hypercoagulable state disease in ALI patients might deteriorate the vascular bed, increase peripheral artery resistance, and lead to poor pedal outflow. Poor pedal run-off vessels in ALI patients have been reported to be a predictor of limb loss (Byrne et al. 2014). Moreover, hypercoagulability has been reported to predict worse outcomes in patients undergoing lower extremity revascularization, including ALI (Torrealba et al. 2019). Thus, hypercoagulable state disease could make revascularisation for ALI difficult, so we need further studies.

An Asian multidisciplinary consensus statement of contemporary CLI suggested that below-the-ankle intervention should only be considered if no clinical improvement with sufficient microcirculation is observed after above-the-ankle intervention (Kawarada et al. 2018). Moreover, several clinical trials have reported that pedal artery disease is an independent predictor of delayed wound healing in CLI patients (Kawarada et al. 2012; Shiraki et al. 2015). Nakama et al. reported that pedal artery angioplasty showed a significantly higher rate of wound healing and a shorter time to wound healing than CLI patients without pedal artery angioplasty (Nakama et al. 2017). Therefore, we speculated that the additional pedal artery angioplasty might be useful for the hypercoagulable state disease patients with CLI subsequently to ALI whose ischemic symptoms do not improve with standard medication (including specialized treatment for hypercoagulable state diseases) and conventional revascularization strategies (surgical, hybrid, or endovascular revascularization). In both cases, angiography revealed the absence of a pedal artery. Balloon angioplasty for BTA based on the angiosome concept was performed, but we found it to be technically and clinically inadequate because of residual severe symptoms in both patients. Therefore, we performed additional pedal artery angioplasty and successfully increased the vascular bed and improved peripheral circulation. Pedal artery angioplasty may be an effective treatment option in symptomatic thrombotic pedal artery occlusion caused by hypercoagulable state diseases.

## Conclusion

We experienced two cases of thrombotic pedal artery occlusion associated with hypercoagulable state diseases in which additional pedal artery angioplasty was effective in improving residual limb ischemia.

## Data Availability

All data generated or analyzed during this study are included in this published article and in its additional files.

## Abbreviations

ALI: Acute limb ischaemia
APS: Antiphospholipid syndrome
ATA: Anterior tibial artery
BTA: Below-the-ankle
CLI: Critical limb ischaemia
DPA: Dorsalis pedis artery
ET: Essential thrombocythemia
PTA: Posterior tibial artery
SPP: Skin perfusion pressure

## References

[CR1] Ali FR, Roberts LN, Mistry H (2009). Recurrent refractory arterial thromboembolism associated with the Janus kinase 2 V617F mutation. J Vasc Surg.

[CR2] Asherson RA, Cervera R, Piette JC, Shoenfeld Y, Asherson RA, Cervera R, Piette JC, Shoenfeld Y (1996). The antiphospholipid syndrome: history, definition, classification, and differential diagnosis. The antiphospholipid syndrome.

[CR3] Byrne RM, Taha AG, Avgerinos E (2014). Contemporary outcomes of endovascular interventions for acute limb ischemia. J Vasc Surg.

[CR4] Chong BK, Mun D, Kang CH (2016). Essential thrombocytosis-associated thromboembolism in the abdominal aorta. Korean J Thorac Cardiovasc Surg.

[CR5] de Donato G, Setacci F, Sirignano P (2014). The combination of surgical embolectomy and endovascular techniques may improve outcomes of patients with acute lower limb ischemia. J Vasc Surg.

[CR6] Fogarty T (2009). Historical reflections on the management of acute limb ischemia. Semin Vasc Surg.

[CR7] Hinojosa CA, Anaya-Ayala JE, Bermudez-Serrato K (2017). Surgical interventions for organ and limb ischemia associated with primary and secondary antiphospholipid antibody syndrome with arterial involvement. Vasc Endovasc Surg.

[CR8] Kawarada O, Fujihara M, Higashimori A (2012). Predictors of adverse clinical outcomes after successful infrapopliteal intervention. Catheter Cardiovasc Interv.

[CR9] Kawarada O, Zen K, Hozawa K (2018). Contemporary critical limb ischemia: Asian multidisciplinary consensus statement on the collaboration between endovascular therapy and wound care. Cardiovasc Interv Ther.

[CR10] Morata Barrado PC, Blanco Cañibano E, García Fresnillo B (2009). Acute lower limb ischemia in a patient with aortic thrombus and essential thrombocytosis. Int J Hematol.

[CR11] Moulinet T, Risse J, Frederic M (2016). Successful treatment with thrombolysis and stent in acute limb ischemia complicating antiphospholipid syndrome. Int J Cardiol.

[CR12] Nakama T, Watanabe N, Haraguchi T (2017). Clinical outcomes of pedal artery angioplasty for patients with ischemic wounds: results from the multicenter RENDEZVOUS registry. JACC Cardiovasc Interv.

[CR13] Patel N, Sacks D, Patel RI (2003). SIR reporting standards for the treatment of acute limb ischemia with use of transluminal removal of arterial thrombus. J Vasc Interv Radiol.

[CR14] Schafer AI (2006). Molecular basis of the diagnosis and treatment of polycythemia vera and essential thrombocythemia. Blood.

[CR15] Shiraki T, Iida O, Takahara M (2015). Predictors of delayed wound healing after endovascular therapy of isolated infrapopliteal lesions underlying critical limb ischemia in patients with high prevalence of diabetes mellitus and hemodialysis. Eur J Vasc Endovasc Surg.

[CR16] Suzuki K, Uemura T, Kikuchi M (2016). Acute limb-threatening ischemia associated with antiphospholipid syndrome: a report of two cases. J Foot Ankle Surg.

[CR17] Torrealba JI, Osman M, Kelso R (2019). Hypercoagulability predicts worse outcomes in young patients undergoing lower extremity revascularization. J Vasc Surg.

